# Genetic variants modulate gene expression statin response in human lymphoblastoid cell lines

**DOI:** 10.1186/s12864-020-06966-4

**Published:** 2020-08-12

**Authors:** Elizabeth Theusch, Yii-Der I. Chen, Jerome I. Rotter, Ronald M. Krauss, Marisa W. Medina

**Affiliations:** 1 Department of Pediatrics, University of California San Francisco, Oakland, CA USA; 2 Department of Pediatrics, The Institute for Translational Genomics and Population Sciences, Los Angeles Biomedical Research Institute at Harbor-UCLA Medical Center, Torrance, CA USA; 3 Departments of Pediatrics and Medicine, The Institute for Translational Genomics and Population Sciences, Los Angeles Biomedical Research Institute at Harbor-UCLA Medical Center, Torrance, CA USA; 4 Departments of Pediatrics and Medicine, University of California San Francisco, Oakland, CA USA

**Keywords:** Statin, Expression quantitative trait locus (eQTL), Gene-environment interaction, RNA-sequencing, Lymphoblastoid cell line

## Abstract

**Background:**

Statins are widely prescribed to lower plasma low-density lipoprotein cholesterol levels. Though statins reduce cardiovascular disease risk overall, statin efficacy varies, and some people experience adverse side effects while on statin treatment. Statins also have pleiotropic effects not directly related to their cholesterol-lowering properties, but the mechanisms are not well understood. To identify potential genetic modulators of clinical statin response, we looked for genetic variants associated with statin-induced changes in gene expression (differential eQTLs or deQTLs) in lymphoblastoid cell lines (LCLs) derived from participants of the Cholesterol and Pharmacogenetics (CAP) 40 mg/day 6-week simvastatin clinical trial. We exposed CAP LCLs to 2 μM simvastatin or control buffer for 24 h and performed polyA-selected, strand-specific RNA-seq. Statin-induced changes in gene expression from 259 European ancestry or 153 African American ancestry LCLs were adjusted for potential confounders prior to association with genotyped and imputed genetic variants within 1 Mb of each gene’s transcription start site.

**Results:**

From the deQTL meta-analysis of the two ancestral populations, we identified significant cis-deQTLs for 15 genes (*TBC1D4*, *MDGA1*, *CHI3L2*, *OAS1*, *GATM*, *ASNSD1*, *GLUL*, *TDRD12*, *PPIP5K2*, *OAS3*, *SERPINB1*, *ANKDD1A*, *DTD1*, *CYFIP2*, and *GSDME*), eight of which were significant in at least one of the ancestry subsets alone. We also conducted eQTL analyses of the endogenous (control-treated), statin-treated, and average of endogenous and statin-treated LCL gene expression levels. We identified eQTLs for approximately 6000 genes in each of the three (endogenous, statin-treated, and average) eQTL meta-analyses, with smaller numbers identified in the ancestral subsets alone.

**Conclusions:**

Several of the genes in which we identified deQTLs have functions in human health and disease, such as defense from viruses, glucose regulation, and response to chemotherapy drugs. This suggests that DNA variation may play a role in statin effects on various health outcomes. These findings could prove useful to future studies aiming to assess benefit versus risk of statin treatment using individual genetic profiles.

## Background

Statins are a class of drugs widely used to lower circulating low-density lipoprotein cholesterol (LDL-C) levels and reduce cardiovascular disease risk [1]. Statins can have other beneficial pleiotropic effects, such as reducing inflammation [2]. However, statin treatment can also have adverse effects, such as myopathy [3] or new-onset diabetes [4]. Though the general mechanism by which statins lower LDL-C is well established [5], there is considerable inter-individual variability in statin efficacy that remains largely unexplained by genetic [6–8] and other [9] factors. In addition, there is much still to be learned about the mechanisms by which statins exert their pleiotropic and adverse effects and how genetic variation impacts statin response at the individual level.

Genetic variants associated with human traits in genome-wide association studies (GWAS) are enriched for those also associated with gene expression levels (expression quantitative trait loci or eQTLs) [10]. Consequently, eQTL datasets contributed by GTEx [11] and others have been instrumental toward improving the annotation of GWAS in recent years, helping to assign candidate causal genes to associated loci. Historically, pharmacogenomics GWAS have not been as well-powered as other traits, since participants need pre-treatment and on-treatment phenotype measurements, limiting the available participant pool [12]. Thus, annotation of sub-genome wide loci from pharmacogenomic GWAS using eQTL data could help to filter signal from noise and identify candidate genes for study.

Environmental exposures can alter the relationships between genetic variants and phenotypes, creating gene-environment interactions (GxE). For instance, drug exposure could differentially change gene expression levels in individuals with different genotypes for a particular “differential eQTL” genetic variant. In contrast to the large number of “endogenous” eQTL studies already conducted in human cells and tissues in their natural, untreated state, there have been a limited number of differential eQTL studies to date, including studies of exposures to immune system stimulation [13–16], UV light [17], drugs [18], or a variety of environmental factors [19] .

In this study, we used a human lymphoblastoid cell line (LCL) statin response model system to identify candidate genetic modulators of clinical statin response. Previous work has demonstrated that statins elicit a strong transcriptional response [20] and that genetic modulators of statin-induced changes in LCL gene structure [21] and expression levels [18] are associated with clinical statin efficacy and adverse events, respectively. Here, we identify additional genetic variants associated with the statin response of clinically important genes that may play a role in statin response.

## Results

### Endogenous eQTLs

We first correlated genetic variation with endogenous gene expression levels to identify eQTLs in 259 Cholesterol and Pharmacogenetics (CAP) European American participant LCLs and in 153 CAP African American LCLs separately. To correct for testing multiple variants per gene, we conducted 100,000 permutations in FastQTL [22]. The most significant eQTL per gene was retained prior to false discovery rate (FDR) adjustment for the number of genes tested. In European American LCLs, 5456 of 13,841 genes tested (39%) were eGenes (genes with at least one significant eQTL) at an FDR of 5% (Additional File 1: Table S1). Similarly, in African American LCLs, 3389 of 13,817 genes tested (25%) were eGenes at an FDR of 5% (Additional File 2: Table S2). Finally, we used METAL [23] to conduct a fixed effects meta-analysis of the eQTL results from the two ethnic subsets, identifying 6065 (44%) eGenes at a threshold of *p* < 1 × 10^− 5^ (Additional File 3: Table S3). Of these, a minority, 104, had a heterogeneity level of significance of *p* < 0.0001, suggesting a difference in effect size between the two ethnic groups.

### Statin-treated eQTLs

**Fig. 1:**
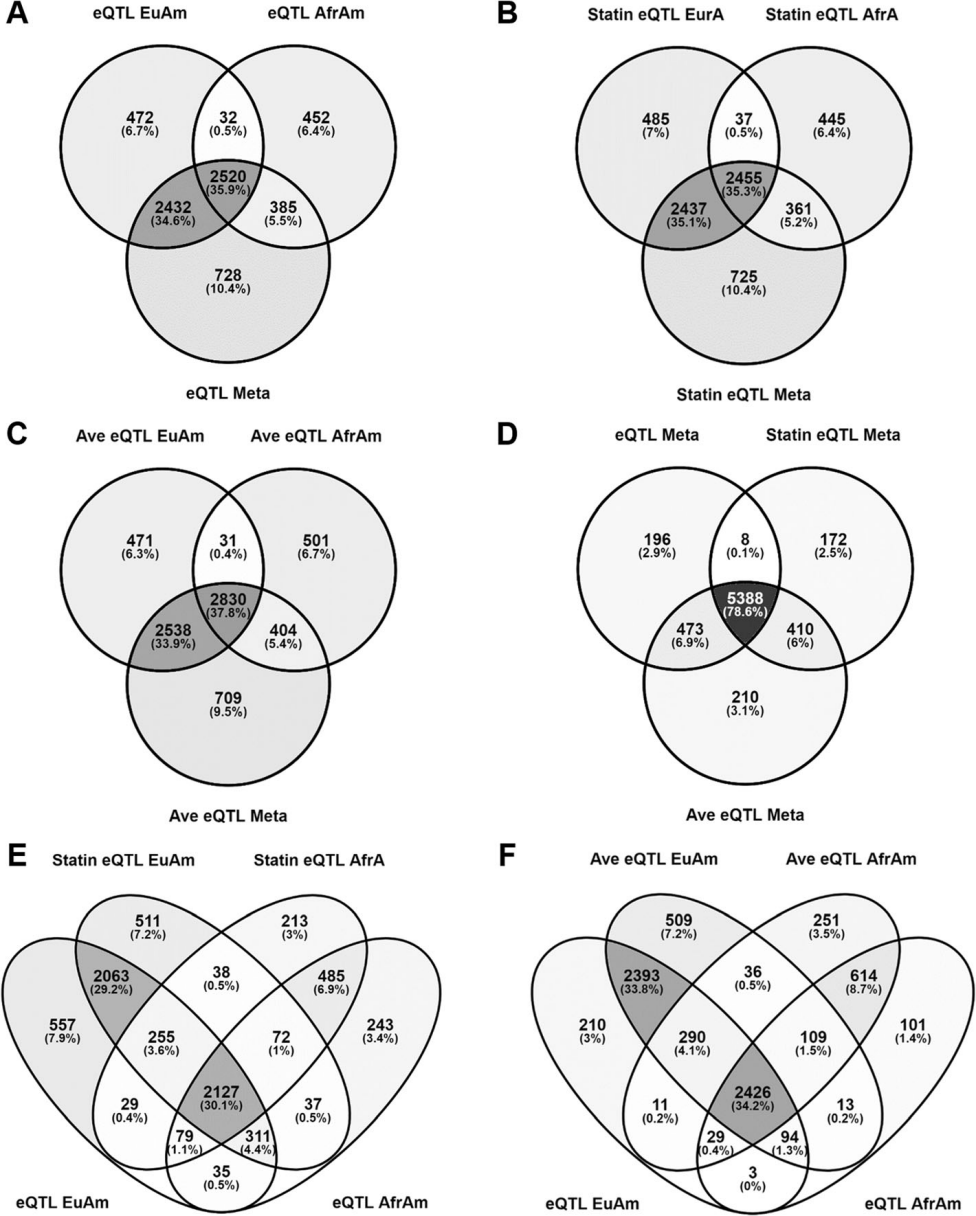
Venn diagrams of overlap between endogenous, statin-treated, and average eQTLs from the meta-analysis and ancestry subsets. Overlap of (**a**) endogenous (**b**) statin-treated and (**c**) average eQTL eGenes between European and African American ancestry subsets and the meta-analysis. The majority of eQTLs identified are not ancestry specific, and the numbers of eGenes identified increases with sample size. **d** Overlap of eGenes from endogenous, statin-treated, and average eQTL meta-analyses. Statin treatment did not significantly change numbers of eGenes, but averaging endogenous and statin-treated expression levels offered increased power for eQTL analysis. Overlap of European and African American ancestry endogenous eQTLs with (**e**) statin-treated and (**f**) average eQTLs. Venn diagrams were created in Venny 2.1 [24]

Similarly, we correlated genetic variation with gene expression levels in statin-treated LCLs. Using this approach, 5414 of 13,841 genes tested (39%) were eGenes (FDR = 5%) in European American LCLs (Additional File 4: Table S4), while 3298 of 13,817 genes tested (24%) were eGenes (FDR = 5%) in African American LCLs (Additional File 5: Table S5). In the meta-analysis of both ethnic subsets, we identified 5978 eGenes (43%) at *p* < 1 × 10^− 5^ (Additional File 6: Table S6). 85 of these were ethnically heterogeneous, with a heterogeneity *p* < 0.0001. Overall, there was a comparable but slightly smaller number of statin-treated eQTLs than endogenous eQTLs identified from the same cell lines. Over 90% of statin-treated eGenes were also endogenous eGenes (Fig. 1 [24]).

### Average eQTLs

Since the majority of eQTL relationships were similar in the endogenous and the statin-treated state in LCLs, we reasoned that averaging control-treated LCL gene expression levels with the corresponding 2 μM simvastatin-treated LCL gene expression levels would increase power to detect eQTLs due to the reduction in measurement error [18]. Indeed, we identified more eQTLs using the average of the control- and statin-treated LCL gene expression levels compared to the control-treated or statin-treated gene expression levels alone. In European American LCLs, we identified 5870 (42%) eGenes with significant average eQTLs (FDR = 5%; Additional File 7: Table S7), while in African American LCLs we identified 3766 (27%) eGenes (Additional File 8: Table S8). In the meta-analysis, there were 6481 (47%) eGenes (*p* < 1 × 10^− 5^), 115 with heterogeneity *p* < 0.0001 (Additional File 9: Table S9). 78.6% of eGenes overlapped between the endogenous, statin-treated, and average eQTL meta-analyses, as shown in Fig. 1 [24].

### European American differential eQTLs

**Fig. 2:**
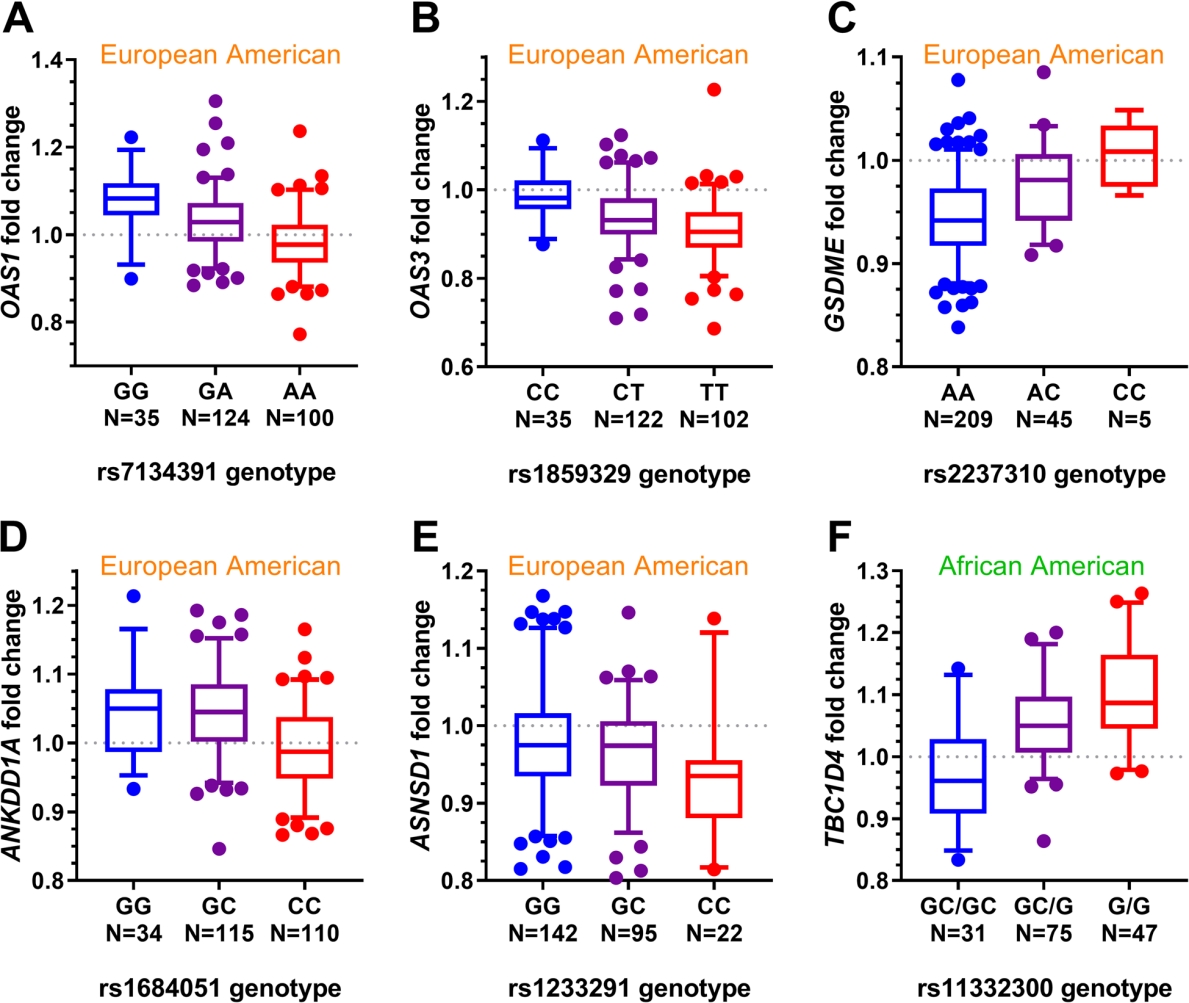
Box and whisker plots of significant deQTL relationships unique to ancestry subsets. **a**-**e** Significant lead deQTLs unique to the European ancestry subset. **f** Significant lead deQTL unique to the African American subset. Genes for which the lead deQTL variant was the same in the European ancestry subset and meta-analysis are shown in Fig. 2 only. In all cases, the reference allele is on the left, in blue. Whiskers mark the 5th and 95th percentiles. Approximate fold changes were calculated by taking 2^(statin-treated minus endogenous variance stabilized gene expression levels) for display purposes

**Fig. 3:**
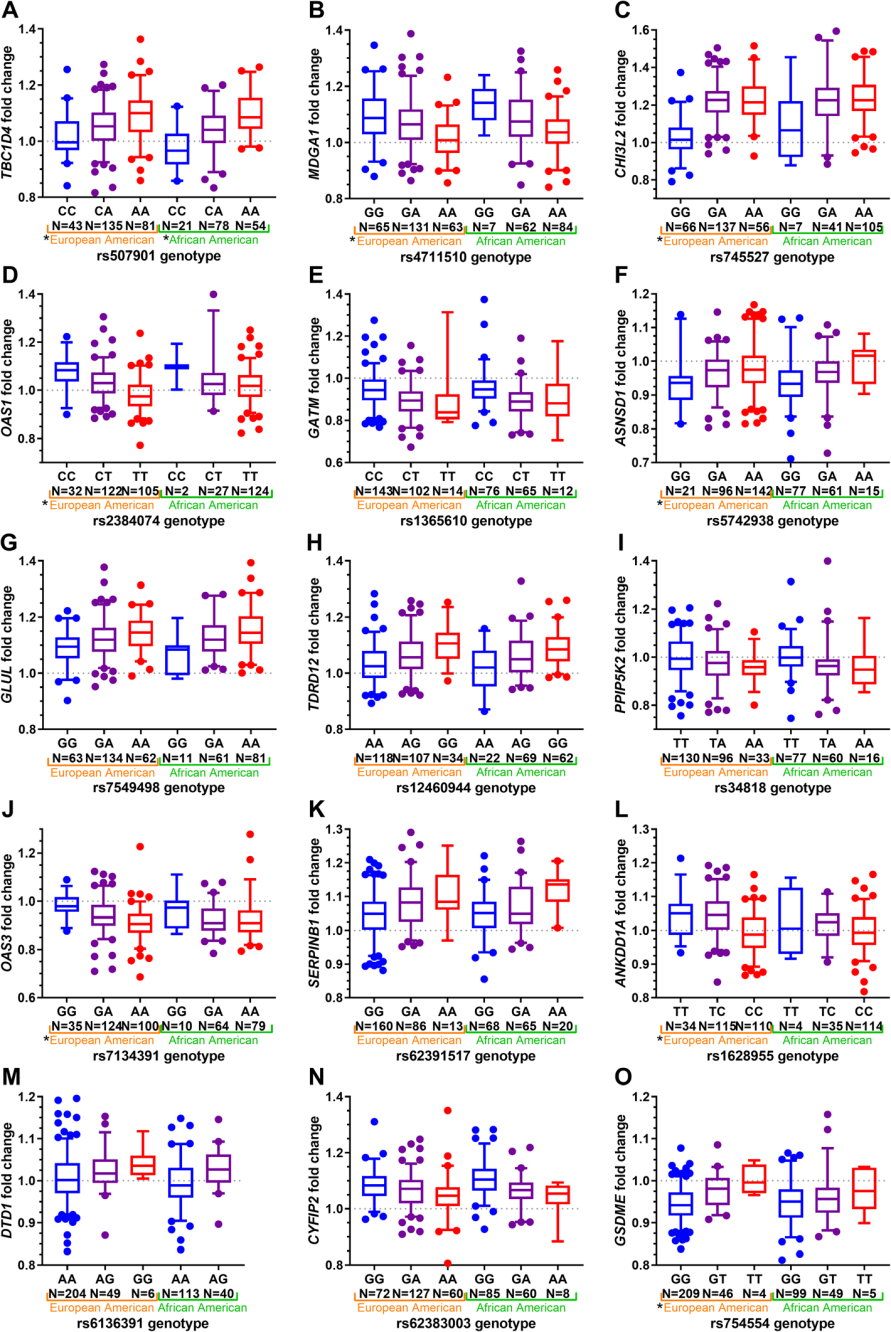
Box and whisker plots of significant deQTLs from meta-analysis. Significant lead deQTLs from meta-analysis are shown in the European (left) and African American (right) subsets in each plot. Asterisks (*) preceding ancestry subset names indicate deQTL relationships that are significant in that ancestry subset. In all cases, the reference allele is on the left, in blue. Whiskers mark the 5th and 95th percentiles. Approximate fold changes were calculated by taking 2^(statin-treated minus endogenous variance stabilized gene expression levels) for display purposes

**Fig. 4:**
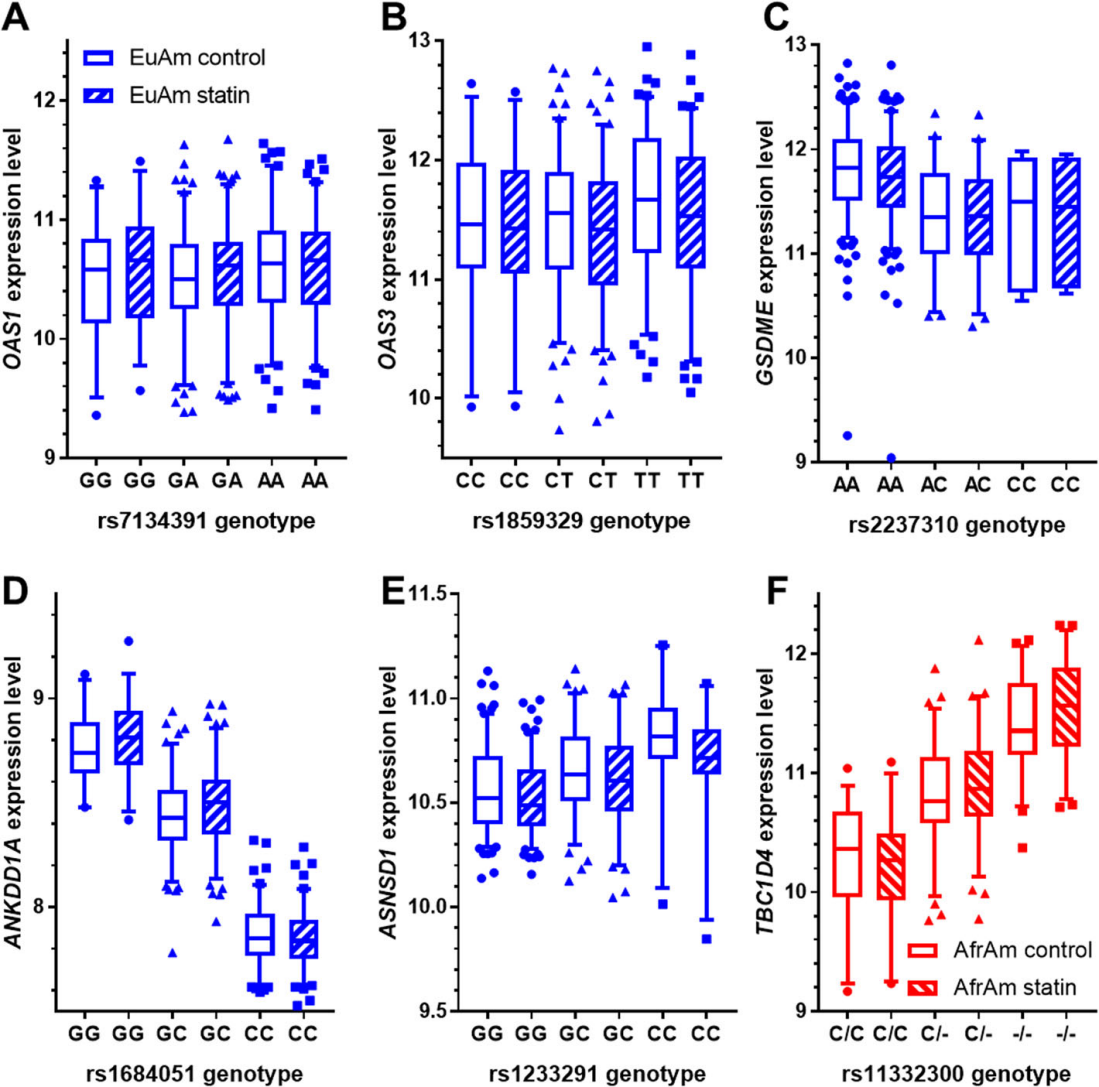
Control and statin-treated gene expression levels split by deQTL genotype for lead deQTLs unique to ancestry subsets. **a**-**e** Significant lead deQTLs unique to the European ancestry subset. **f** Significant lead deQTL unique to the African American subset. Genes for which the lead deQTL variant was the same in the European ancestry subset and meta-analysis are shown in Fig. 5 only. In all cases, the reference allele is on the left. Whiskers mark the 5th and 95th percentiles. Approximate fold changes were calculated by taking 2^(statin-treated minus endogenous variance stabilized gene expression levels) for display purposes. Sample sizes match those in Fig. 2

**Fig. 5:**
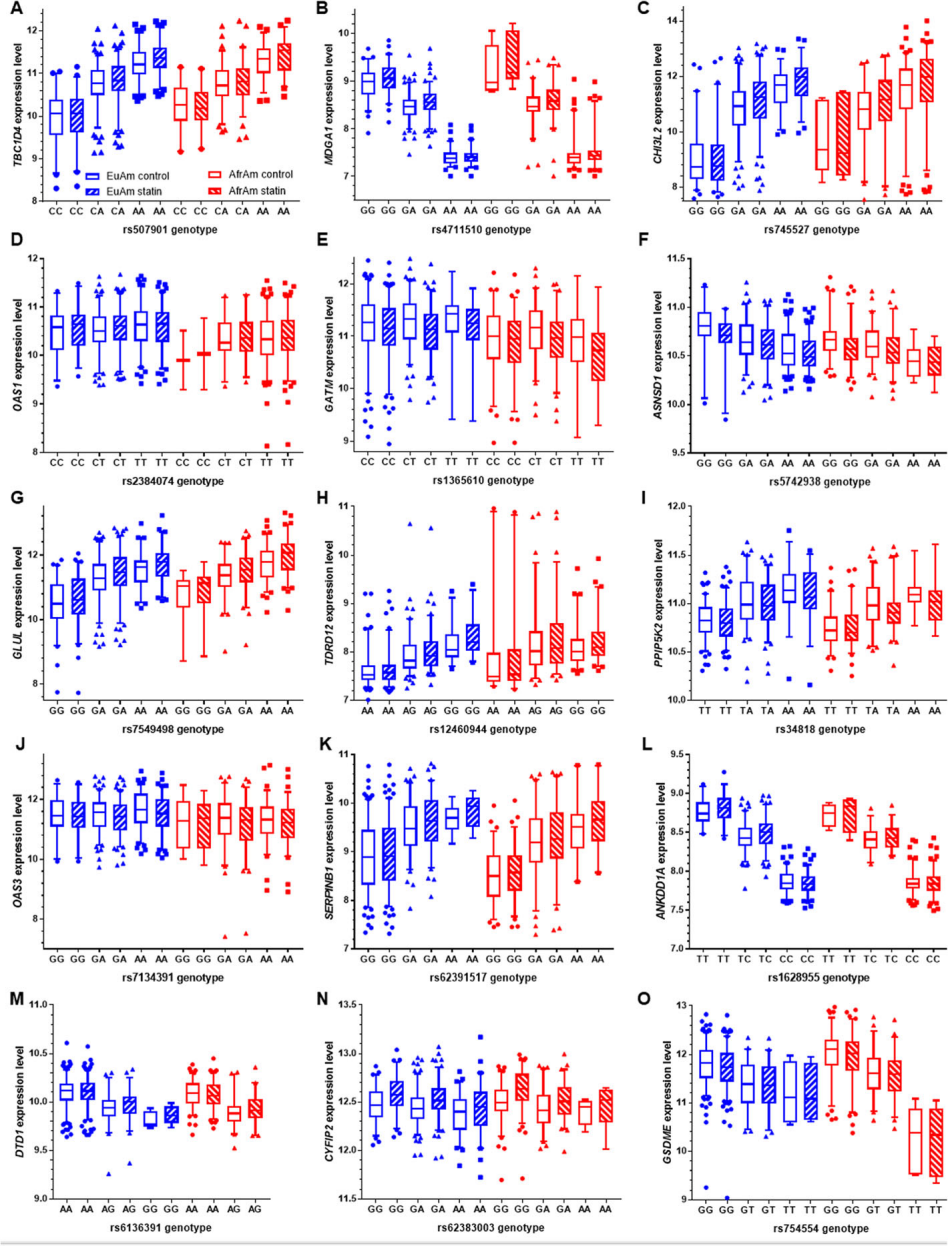
Control and statin-treated gene expression levels split by deQTL genotype for lead deQTLs from meta-analysis. Gene expression levels of **a** *TBC1D4* **b** *MDGA1* **c** *CHI3L2* **d** *OAS1* **e** *GATM* **f** *ASNSD1* **g** *GLUL* **h** *TDRD12* **i** *PPIP5K2* **j** *OAS3* **k** *SERPINB1* **l** *ANKDD1A* **m** *DTD1* **n** *CYFIP2* or **o** *GSDME* genes are plotted split by their significant lead deQTL genotypess from the meta-analysis in the European (left) and African American (right) subsets in each plot. In all cases, the reference allele is on the left. Whiskers mark the 5th and 95th percentiles. Approximate fold changes were calculated by taking 2^(statin-treated minus endogenous variance stabilized gene expression levels) for display purposes. Sample sizes match those in Fig. 3

Using statin-induced changes in LCL gene expression and imputed genotype data from 259 CAP participants of European ancestry, we identified genetic variants significantly associated with the statin response (i.e. change in transcript levels) of eight genes (FDR 5%; Table 1**,** Fig. 2a-e & Fig. 3a-c). For six of these genetic variants, the endogenous eQTL relationship with gene expression levels was stronger than the differential eQTL relationship. For the remaining two deQTL genes, *OAS1* and *OAS3,* we did not identify a strong endogenous eQTL in our dataset (Fig. 4a-e **&** Fig. 5a-c). However, other studies have reported all eight of the deQTL variants to be eQTL variants in at least one cell or tissue type (Additional File 10: Table S10) [11, 25, 26].

**Table 1:** Significant lead deQTLs in European ancestry LCLs

Variant	Chr	BP	Gene	|Dist. to TSS| (bp)	Ref	Alt	Alt Freq.	Effect Size (Alt)	Nominal P	Beta Perm Q
rs745527	1	111,745,975	*CHI3L2*	2581	G	A	48%	0.73	4.8E-16	8.50E-08
rs7134391	12	113,366,691	*OAS1*	22,108	G	A	63%	−0.73	1.9E-14	7.10E-07
rs1859329	12	113,376,452	*OAS3*	294	C	T	63%	−0.65	6.7E-12	9.90E-05
rs507901	13	75,869,652	*TBC1D4*	186,599	C	A	57%	0.63	7.3E-11	9.50E-04
rs4711510	6	37,669,641	*MDGA1*	2558	G	A	50%	−0.54	3.5E-10	0.0034
rs2237310	7	24,759,648	*GSDME*	49,597	A	C	11%	0.81	3.0E-10	0.0034
rs1684051	15	65,198,309	*ANKDD1A*	5793	G	C	65%	−0.54	2.0E-09	0.006
rs1233291	2	190,690,951	*ASNSD1*	164,839	G	C	27%	−0.57	6.2E-09	0.016

**Fig. 6:**
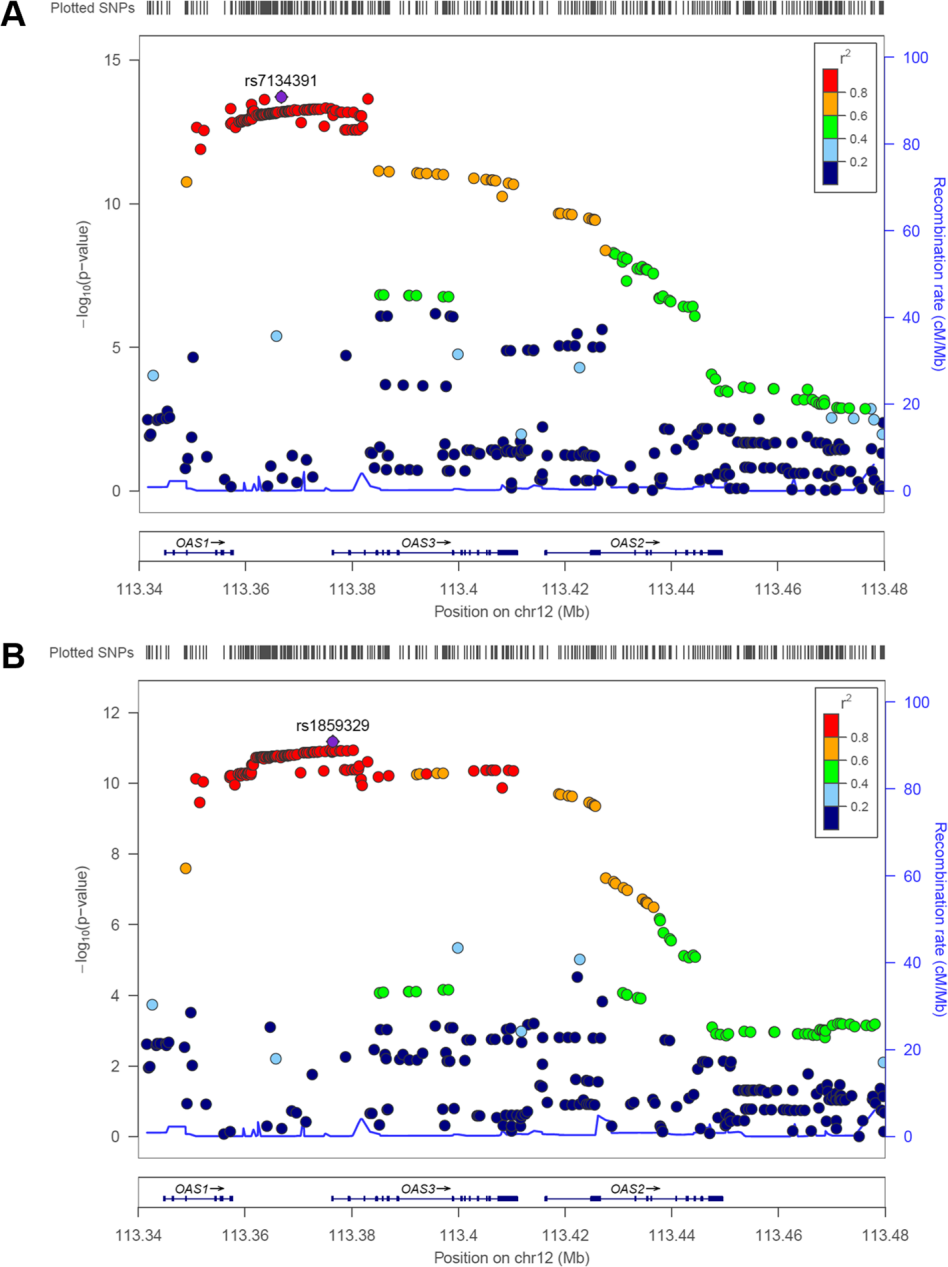
LocusZoom plots of (**a**) *OAS1* and (**b**) *OAS3* deQTL associations in European ancestry subset. Linkage disequilibrium information is derived from EUR individuals in the November 2014 1000 Genomes data release

**Fig. 7:**
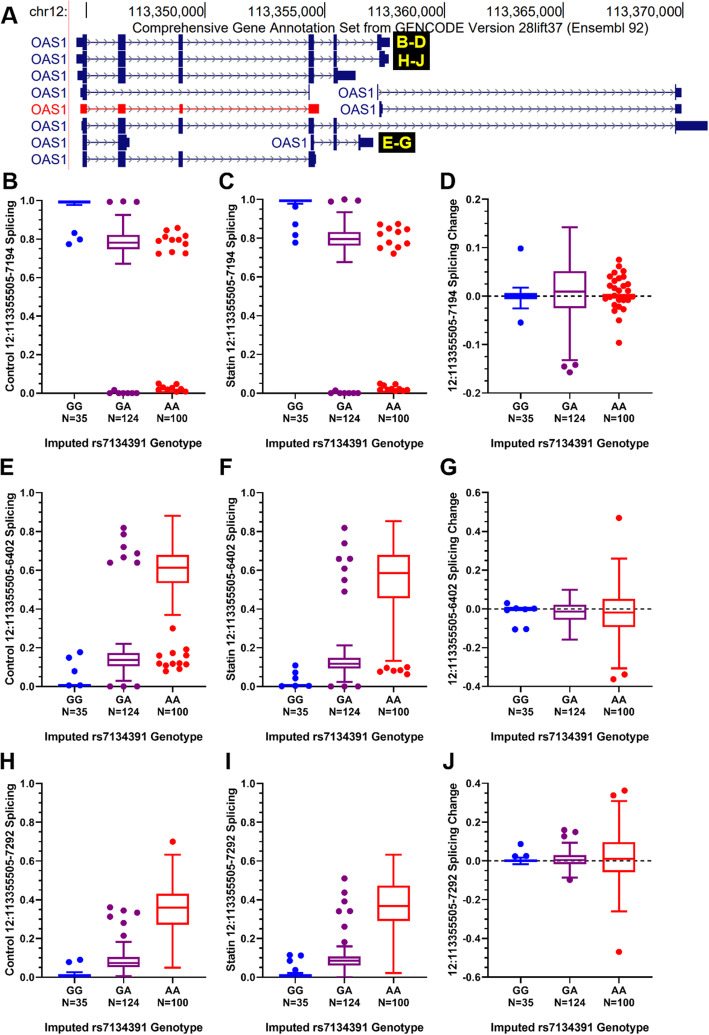
Endogenous, statin-treated, and statin-induced changes in *OAS1* splicing by deQTL genotype in European ancestry subset. **a** *OAS1* annotated isoforms. For three abundant splice isoforms with introns beginning at hg19 chr12:113355505 (p46:**b**-**d**, unnamed isoform: **e**-**g**, p48:**h**-**j**), the proportion of each isoform in control- and statin-treated European ancestry LCLs was calculated using junction-spanning read fractions. Isoform fractions in (B,E,H) control- and (C,F,I) statin-treated LCLs as well as the (D,G,J) statin-induced changes in these isoform fractions were plotted by European ancestry lead *OAS1* deQTL (rs7134391) imputed genotypes in Tukey box and whisker plots. Since genotypes were imputed, some of the outlying points may be genotype assignment errors

*OAS1* and *OAS3* are adjacent to each other in the genome, and their lead deQTL variants are in strong linkage disequilibrium (r^2^ = 0.97) in EUR (Fig. 6) [27]. They are also in LD with a known *OAS1* splice site mutation (rs10774671) in EUR (r^2^ = 0.88) but not AFR (r^2^ < 0.2) and were strongly (*p* < 10^− 50^) correlated with endogenous, but not statin-induced changes in, *OAS1* splicing in the CAP European American LCLs (Fig. 7).

### African American differential eQTL

**Fig. 8:**
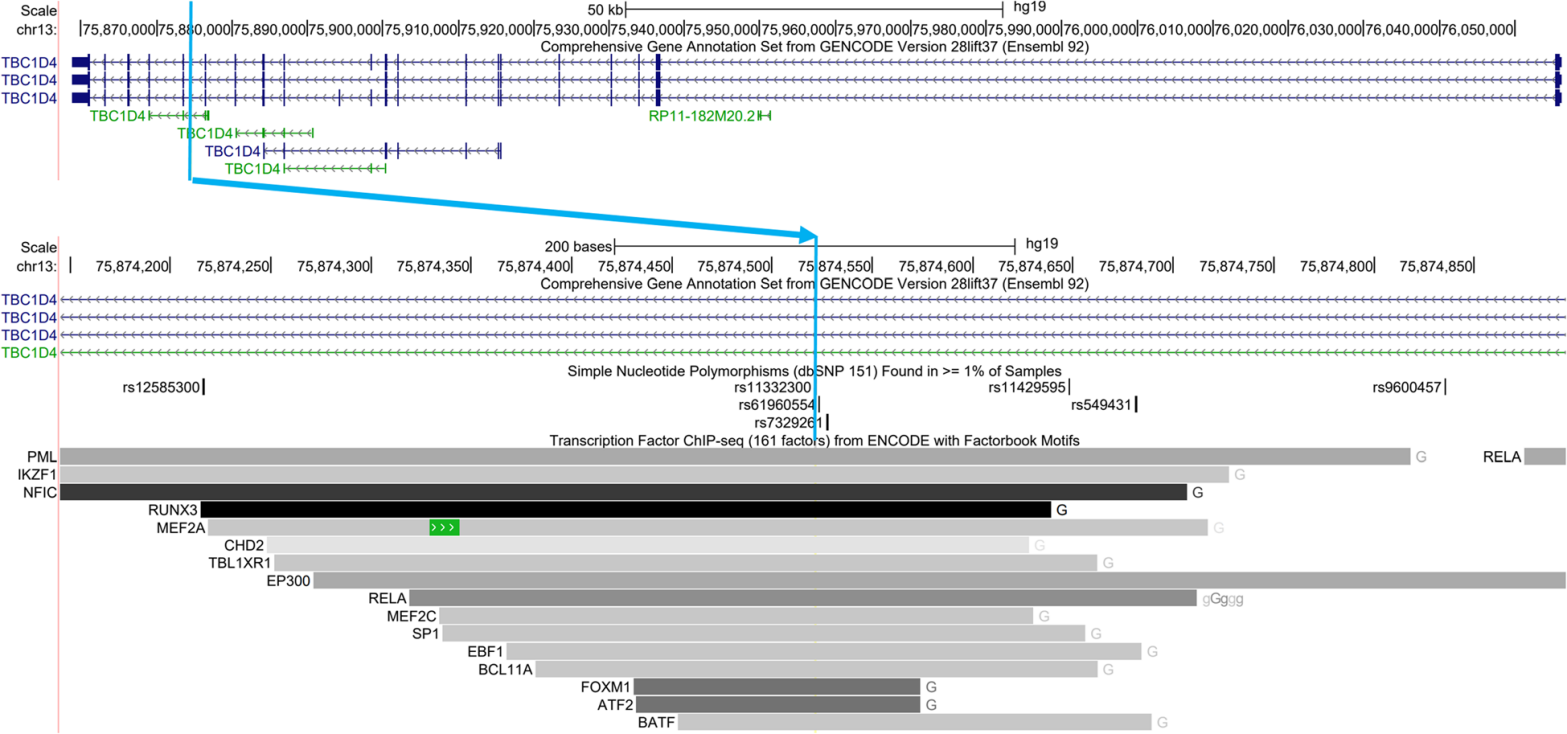
Regulatory elements overlapping *TBC1D4* lead deQTL from African American ancestry subset

Similarly, we conducted a deQTL analysis in 153 CAP participants of African American ancestry. *TBC1D4* was the only gene with a significant (FDR = 5%) deQTL in this population, and its most significant deQTL was rs11332300 (Table 2**,** Fig. 2f). Interestingly, rs11332300 and adjacent variants rs61960554 and rs7329261 in strong linkage disequilibrium (r^2^ > 0.98 in AFR and EUR) may be functional variants because they are located in a LCL enhancer element based on ChIP-seq data from the ENCODE project (Fig. 8) [28, 29]. These variants are also endogenous eQTLs for *TBC1D4*(Additional File 10: Table S10) [11, 25, 26]. These three variants were not imputed in the CAP European Americans using the Haplotype Reference Consortium reference panel, but the fourth most significant variant in more modest LD (r^2^ = 0.58 in AFR and 0.92 in EUR) with the first three, rs507901, was found in both datasets.

**Table 2:** Top deQTLs in African American ancestry LCLs

Variant	Chr	BP	Gene	|Dist. to TSS| (bp)	Ref	Alt	Alt Freq.	Effect Size (Alt)	Nominal P	Beta Perm Q
rs11332300	13	75,874,521	*TBC1D4*	181,730	GC	C	55%	0.77	1.41E-12	2.6E-04
rs61960554	13	75,874,524	*TBC1D4*	181,727	T	C	55%	0.77	1.47E-12	N/A
rs7329261	13	75,874,528	*TBC1D4*	181,723	T	C	55%	0.77	1.47E-12	N/A
rs507901	13	75,869,652	*TBC1D4*	186,599	C	A	60%	0.71	1.18E-09	N/A

### Differential eQTL meta-analysis

Significant deQTLs for 15 genes were identified in the meta-analysis (*p* < 5 × 10^− 9^; Table 3**,** Fig. 3), and these included deQTLs for all eight of the genes identified in the ethnic subset analyses. All of these variants were within 200 kb of their target genes, though the search space was five times larger. Four of the 15 significant deQTL loci (*CHI3L2*, *OAS1*, *OAS3*, and *GSDME*) showed evidence for ethnic heterogeneity in effect sizes, with stronger effects seen in individuals of European ancestry.

**Table 3:** Lead deQTLs from meta-analysis with heterogeneity statistics

Marker	Chr	BP	Gene	|Dist. to TSS| (bp)	Alt Dir	Meta P	EurAm P	AfrAm P	Het ISq	Het PVal
rs507901	13	75,869,652	*TBC1D4*	186,599	++	7.3E-19	7.3E-11	1.2E-09	0	0.39
rs4711510	6	37,669,641	*MDGA1*	2558	–	2.1E-14	3.5E-10	1.2E-05	0	0.73
rs745527	1	111,745,975	*CHI3L2*	2581	++	3.0E-13	4.8E-16	0.16	93.2	1.3E-4
rs2384074	12	113,382,977	*OAS1*	38,394	–	9.5E-13	2.2E-14	0.076	90.5	1.2E-3
rs1365610	15	45,694,610	*GATM*	84	–	1.1E-12	4.8E-07	3.1E-07	0	0.32
rs5742938	2	190,649,958	*ASNSD1*	123,846	++	1.1E-11	7.2E-09	2.9E-04	0	0.51
rs7549498	1	182,363,623	*GLUL*	2281	++	2.2E-11	3.9E-06	6.5E-07	22.1	0.26
rs12460944	19	33,305,725	*TDRD12*	95,065	++	2.5E-11	7.3E-08	7.9E-05	0	0.88
rs34818	5	102,436,111	*PPIP5K2*	19,743	–	2.7E-11	1.2E-07	5.2E-05	0	0.98
rs7134391	12	113,366,691	*OAS3*	9467	–	2.1E-10	1.9E-11	0.092	86.8	5.9E-3
rs62391517	6	2,936,204	*SERPINB1*	93,963	++	3.7E-10	1.8E-06	4.8E-05	0	0.76
rs1628955	15	65,187,220	*ANKDD1A*	16,882	–	1.2E-09	2.7E-09	0.025	70.8	6.4E-2
rs6136391	20	18,480,845	*DTD1*	87,693	++	1.6E-09	1.1E-05	2.9E-05	0	0.53
rs62383003	5	156,700,461	*CYFIP2*	7371	–	2.2E-09	8.3E-06	5.9E-05	0	0.64
rs754554	7	24,758,818	*GSDME*	50,427	++	4.8E-09	4.0E-10	0.14	85.7	8.1E-3

All 15 variants were also eQTLs for the deQTL genes in at least one cell or tissue type (Additional File 10: Table S10) [11, 25, 26], but the deQTLs for *OAS1*, *OAS3* and *GATM* were not significant eQTLs in the CAP LCLs from our study (Table 4, Fig. 5). Though statin treatment enhanced the eQTL relationship for most deQTLs (8 out of 12), it dampened the eQTL relationship for others (e.g. *ASNSD1*), which is reflected in the weaker statin eQTL association compared to the eQTL association (Table 4) and the different directionality of the deQTL and eQTL associations (Figs. 3 & 5). For example, the association of rs5742938 genotype with *ASNSD1*statin-treated expression levels was more modest (*P* = 1.6 × 10^− 26^) than its association with endogenous expression levels (*P* = 2.1 × 10^− 47^) (*p* = 0.04 and *p* = 0.08 for difference between endogenous and statin eQTL correlations using Fisher’s r to z transformation in European and African American subsets, respectively), and the alternate “C” allele of rs5742938 was correlated with higher endogenous and statin-treated*ASNSD1* levels but with greater reductions in *ASNSD1* expression with statin treatment.

**Table 4:** eQTL, statin eQTL, and average eQTL relationships of deQTL associations

Marker	Gene	deQTL Meta Dir	deQTL Meta P	eQTL Meta Dir	eQTL Meta P	Statin eQTL Meta Dir	Statin eQTL Meta P	Ave eQTL Meta Dir	Ave eQTL Meta P
rs507901	*TBC1D4*	++	7.30E-19	++	3.02E-55	++	3.30E-60	++	9.41E-58
rs4711510	*MDGA1*	–	2.10E-14	–	2.02E-102	–	8.09E-101	–	3.41E-102
rs745527	*CHI3L2*	++	3.00E-13	++	1.68E-29	++	3.07E-30	++	5.64E-30
rs2384074	*OAS1*	–	9.50E-13	++	9.73E-02	−+	2.81E-01	−+	8.41E-01
rs1365610	*GATM*	–	1.10E-12	++	1.26E-03	++	3.38E-01	++	3.82E-02
rs5742938	*ASNSD1*	++	1.10E-11	–	2.11E-47	–	1.64E−26	–	5.35E-40
rs7549498	*GLUL*	++	2.20E-11	++	1.58E-29	++	4.31E-31	++	1.45E-30
rs12460944	*TDRD12*	++	2.50E-11	++	4.12E-27	++	1.73E-31	++	1.17E-29
rs34818	*PPIP5K2*	–	2.70E-11	++	1.28E-76	++	2.49E-65	++	7.48E-73
rs7134391	*OAS3*	–	2.10E-10	++	2.29E-03	++	1.07E-01	++	1.95E-02
rs62391517	*SERPINB1*	++	3.70E-10	++	1.85E-38	++	2.39E-42	++	6.01E-41
rs1628955	*ANKDD1A*	–	1.20E-09	–	6.99E-97	–	6.05E-97	–	9.30E-98
rs6136391	*DTD1*	++	1.60E-09	–	5.89E-37	–	3.09E-25	–	1.06E-32
rs62383003	*CYFIP2*	–	2.20E-09	–	6.84E-08	–	2.43E-13	–	1.11E-10
rs754554	*GSDME*	++	4.80E-09	–	8.85E-36	–	1.70E-33	–	1.09E-34

**Fig. 9:**
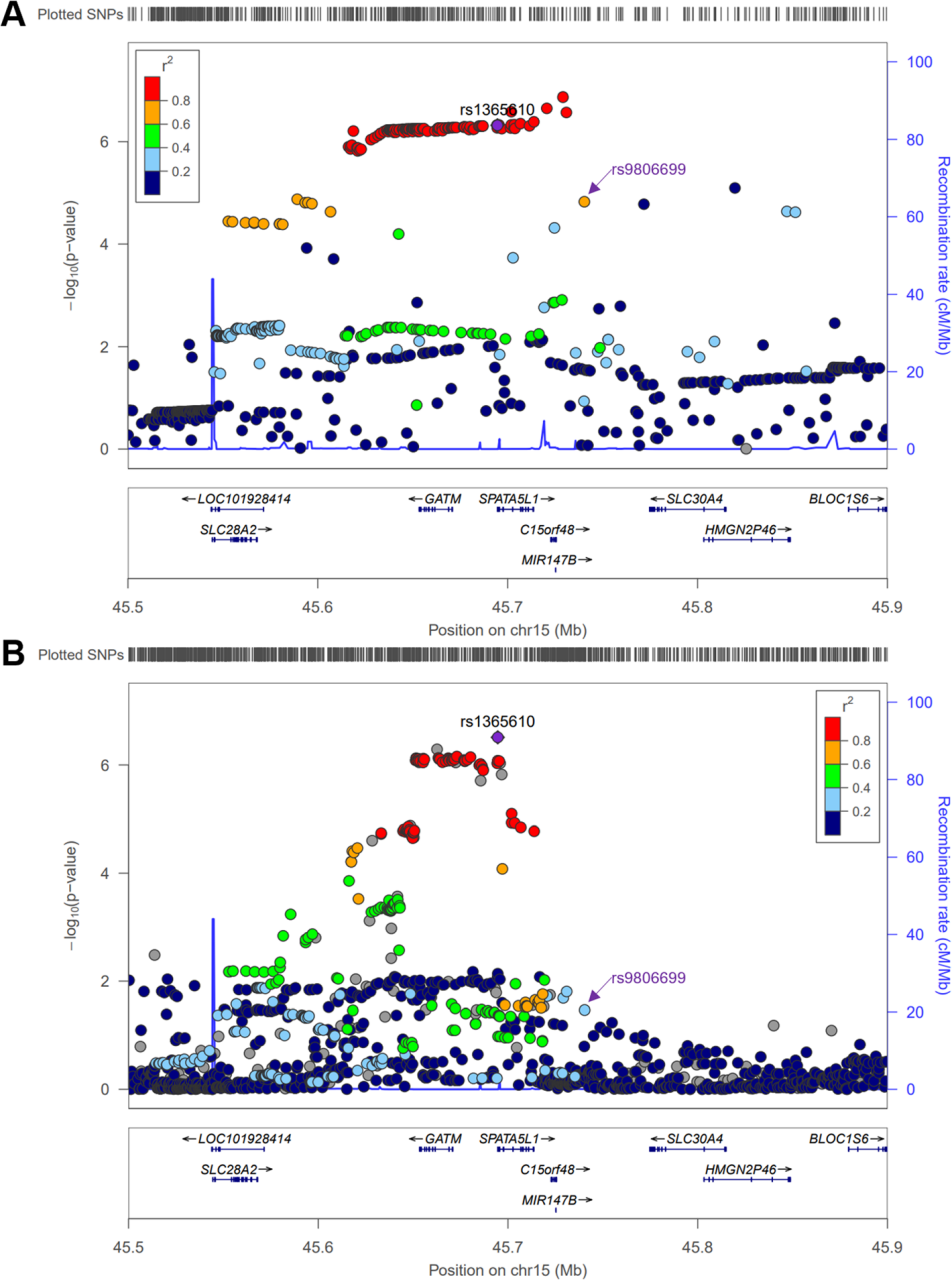
LocusZoom plots of *GATM* deQTL results in each ancestry subset. **a** European ancestry deQTL using LD information from November 2014 1000 Genomes EUR (**b**) African American ancestry deQTL using LD information from November 2014 1000 Genomes AFR

**Fig. 10:**
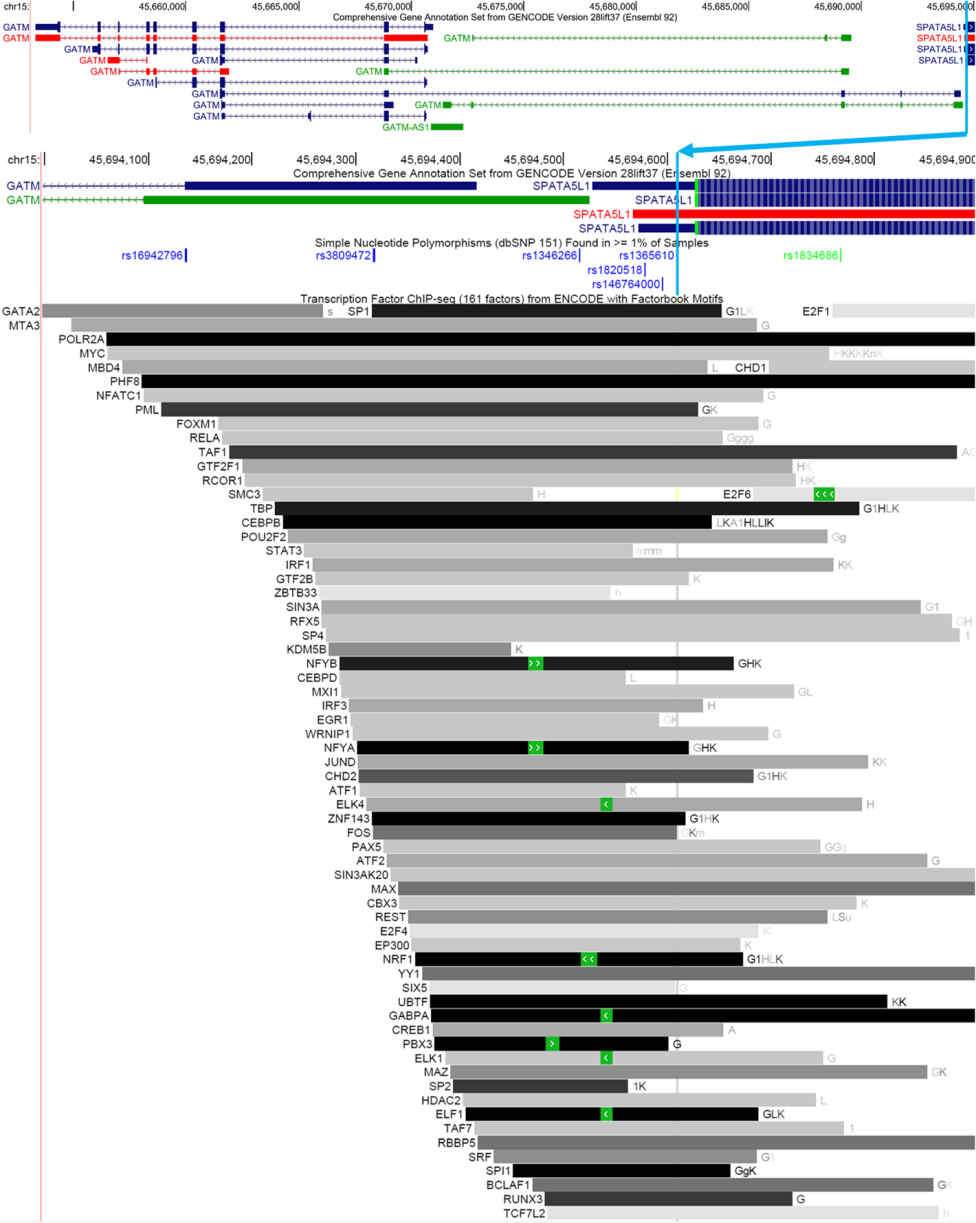
Regulatory elements overlapping *GATM* lead deQTL from meta-analysis

Of note, a deQTL for *GATM* was previously identified using gene expression array data from a partially overlapping sample set of 480 European American CAP LCLs [18]. (211 European ancestry LCLs were used in both studies.) The lead deQTL variant in that study, rs9806699, is in moderately strong linkage disequilibrium with our lead deQTL variant, rs1365610, in EUR (r^2^ = 0.768) but not AFR (r^2^ = 0.266) populations [27] (Fig. 9). rs1365610 could itself be a functional variant based on its location in a collection of regulatory elements just upstream of an alternate *GATM* first exon (Fig. 10) [28, 29].

**Fig. 11:**
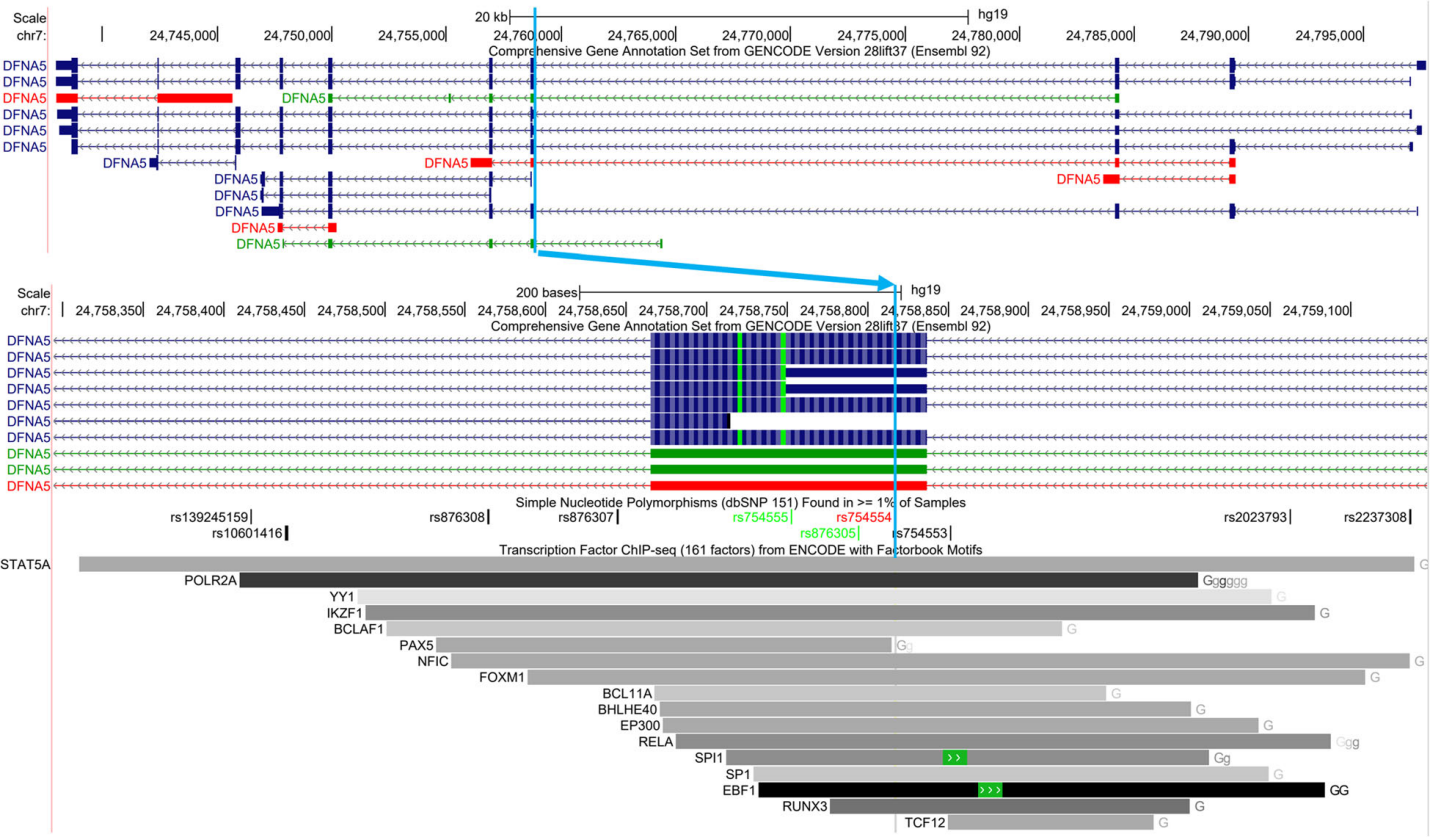
Regulatory elements overlapping *GSDME* (aka *DFNA5*) lead deQTL from meta-analysis

The lead deQTL for *GSDME* (aka *DFNA5*), rs754554, is a missense variant that is located in an LCL enhancer element based on ENCODE ChIP-seq data (Fig. 11) [28, 29], while the lead deQTL variant from the European American subset analysis, rs2237310, lacks this level of support for functionality. This is also reflected by an improved RegulomeDB score for rs754554 (score of 1f reflecting a likelihood that the variant affects transcription factor binding and is an eQTL) compared to rs2237310 (score of 6 showing minimal binding evidence), a phenomenon that was also observed for lead deQTL variants for other genes, such as *ANKDD1A* (score of 1f for rs1628955 from meta-analysis versus 5 for rs1684051 from European ancestry subset) [30]. Similar to the *GATM* deQTL results, this illustrates the utility of adding the African American to the European American data.

## Discussion

In this study, we identify genetic variants (“differential eQTLs”) that influence the statin response of 15 genes in lymphoblastoid cell lines from European American and African American CAP study participants. We also detect endogenous, statin-treated, and average eQTLs for about 6000 genes in the same population of LCLs.

In our eQTL analyses, we identify substantially more eGenes than a study [25] of a comparable number of LCLs (> 6000 vs. < 4000 eGenes identified) and we identify approximately the proportion of eGenes observed in GTEx tissues with this sample size [11], demonstrating the robustness of our data and analysis methods. In addition, the average eQTL analysis yields an additional 620 associations that are not identified by the endogenous eQTL meta-analysis, illustrating the utility of repeat measures to increase power when sample sizes are limited. Furthermore, 104 of the endogenous eQTLs we identify in the trans-ethnicmeta-analysis have heterogeneous effects between LCLs of European and African American ancestry, indicating a small subset of endogenous eQTL relationships exhibit ethnic heterogeneity..

The 15 deQTLs all share several properties that support their validity. First, they are close to the transcription start site of the target gene. Despite considering all variants 1 Mb up- and downstream of the TSS, they are all within 200 kb. Second, they all show consistent directionality between the European American and African American ethnic groups, though there is some heterogeneity in effect sizes between ethnic groups for four of the deQTLs. Third, they are all also endogenous eQTLs in at least one cell or tissue type in published studies [11, 25, 26]. Most, but not all, are endogenous eQTLs in the CAP LCLs.

In contrast to the thousands of eQTLs we identify in these 412 LCLs, we only find a modest 15 deQTLs. This highlights the large sample size necessary to detect relatively subtle GxE interactions with sufficient power. The modest number of deQTLs identified is also consistent with the handful of associated genetic loci identified in GWAS studies of lipid statin response [8] compared to the hundreds of loci associated with plasma lipids to date [31, 32]. Together, these findings suggest that the DNA-drug interaction might only be a minor contributor to inter-individual variation in drug response.

Some of the deQTL findings may be specific to LCLs or blood, while others are likely to be more broadly relevant to other tissues. Most of the deQTL variants have been reported to be endogenous eQTLs in multiple tissues, but others (such as the *TBC1D4* deQTL) seem to be more narrowly restricted to LCLs or blood. This is exemplified by the fact that some deQTL variants, such as the *GATM* deQTL, fall within regulatory elements in multiple cell types based on ChIP-seq data from the ENCODE project [28], while others may be restricted to regulatory elements active only in LCLs or similar blood cell types. Statins affect a wide-range of tissues, as evidenced by both the pleiotropy of statin benefit (i.e. reduction of circulating cholesterol and anti-inflammatory properties), as well as the broadness of statin adverse effects (i.e. statin-induced diabetes, myopathy, etc.). Thus, the tissue-specificity (or lack of specificity) of individual deQTLs may be used to infer which (if any) of these statin effects they may impact.

Unsurprisingly, with the increased sample size in the meta-analysis, we discover more deQTLs than in the ethnic subset analyses. Importantly, we also observe a greater increase in precision of deQTL association signals in the meta-analysis compared to the European American subset analysis, which could be due in part to the reduced linkage disequilibrium between loci in the African American population [33], even though there are a relatively modest number of African Americans included in this study. In fact, the lead deQTL variants for *GATM*, *GSDME*, and *ANKDD1A* from the meta-analysis could themselves be functional since regulatory elements overlap their positions, unlike the corresponding lead deQTL variants from the European ancestry subset analysis.

Using a partially overlapping set of CAP European American LCLs, a deQTL was previously identified for glycine amidinotransferase (*GATM*), a creatine synthesis enzyme [18]. Here we show that the published *GATM* deQTL (rs9806699) is less likely to be functional than the lead deQTL variant (rs1365610) identified in the current analysis. Not only is rs1365610 located in a regulatory element just 84 bp upstream of an alternate *GATM* TSS, but it is also the strongest *GATM* deQTL in the African American LCLs, while the rs9806699 association with *GATM* statin response is only observed in European American LCLs.

In addition to *GATM*, which encodes a metabolically important enzyme, we identify deQTLs in other clinically significant genes. For instance, given reports that statins increase diabetes risk [4, 34, 35], it is interesting that we identify a deQTL for TBC1 domain family member 4 (*TBC1D4*), a gene which plays a role in glucose homeostasis and type 2 diabetes [36–38]. In addition, cellular gasdermin E (*GSDME/DFNA5*) expression levels help determine the type of cell death (i.e. pyroptosis or apoptosis) that is stimulated by chemotherapy drugs, with *Gsdme* knockout mice experiencing less side effects from the chemotherapy drug cisplatin than their wild type counterparts [39]. This is particularly interesting given that epidemiological studies have suggested that statin use reduces cancer-related mortality [40], and statins are well known to be cytotoxic in cellular models (reviewed in [41]), at least at supraphysiological levels.

2′-5′-oligoadenylate synthetase 1 (*OAS1*) and 2′-5′-oligoadenylate synthetase 3 (*OAS3*) are important components of the innate immune system that are induced by interferon and can activate RNase L, which in turn can degrade cellular and viral RNAs and impair viral replication. Due to this activity, *OAS1* has been a target of intense natural selection in humans [42]. Alternative splicing of *OAS1* results in several isoforms with different enzymatic activity [43], and the splice site polymorphism rs10774671 has been associated with infection by viruses [44]. There is some evidence that statins have anti-viral activity against viruses such as Hepatitis C [45, 46], HIV-1 [47], poliovirus [48], and cytomegalovirus [49], though the mechanism(s) involved are poorly understood. In the future, it would be interesting to investigate whether the antiviral activity of statin treatment differs by *OAS1/3* deQTL genotype.

## Conclusions

Overall, this study provides a resource of eQTLs identified in European ancestry and African American ancestry cell lines and identifies genetic variants that modulate the statin response of some clinically interesting genes. In the future, these differential eQTL variants could be incorporated into panels designed to predict benefits versus risk of statin therapy.

## Methods

### Participants and genotyping

This study uses genome-wide genotype and lymphoblastoid cell line (LCL) transcriptomic data derived from 412 of 944 Cholesterol and Pharmacogenetics (CAP) 40 mg/day 6 week simvastatin clinical trial participants (ClinicalTrials.gov ID: NCT00451828) [9]. Demographic and phenotypic characteristics of this participant subset are shown in Table 5. Self-reported white CAP participants were genotyped as previously described on one or more (Illumina HumanHap300, Human610-Quad, custom iSelect and Cardio-Metabochip) platforms [50, 51], and self-reported black participants were genotyped on the Illumina HumanOmni2.5Exome and, for the majority of participants, the Cardio-Metabochip and Immunochip.

**Table 5:** Characteristics of European ancestry and African American ancestry CAP participants used in eQTL analyses

	African Americans	European Americans
N	153	259
Gender	53.6% Female	47.9% Female
Age (years)	53.7 ± 12.8	54.2 ± 12.0
BMI	30.1 ± 6.0	27.9 ± 5.7
Smoker	28.8%	10.8%
^a^Total Cholesterol (mg/dl)	204 ± 36	214 ± 37
^a^LDL Cholesterol (mg/dl)	129 ± 35	135 ± 33
^a^HDL Cholesterol (mg/dl)	55 ± 17	54 ± 17
^a^Triglycerides (mg/dl)	103 ± 49	125 ± 67
% change TC	-26 ± 10%	−28 ± 9%
% change LDLC	−40 ± 13%	−43 ± 11%
% change HDLC	2 ± 11%	5 ± 11%
% change TG	−14 ± 26%	−17 ± 24%

Values are mean ± SD. ^a^Values are prior to the start of statin treatment

### Genotype imputation

Prior to imputation, 3 sex mismatches, 2 related individuals, and 5 ancestry outliers were excluded from the subset of 422 CAP participants with both RNA-seq and genome-wide genotype data, and participants were divided into two groups (European American and African American) based on their genetic ancestry. Monomorphic, multi-allelic, and multi-mapping markers were excluded, except those that mapped to the X and Y chromosomes. Markers with greater than 5% missingness or with significant deviations from Hardy-Weinberg equilibrium (*p* < 0.000001 or *p* < 0.00001 for European and African Americans, respectively) were also excluded. European Americans were imputed with the Haplotype Reference Consortium v1.1 reference panel [52] using minimac3 on the Michigan Imputation server [53]. African Americans were imputed with the 1000 genomes phase3v5 cosmopolitan reference panel [27] using MaCH Admix [54].

### RNA sequencing and analysis

LCLs were established through Epstein-Barr virus (EBV) transformation of blood sample-derived lymphocytes as previously described [18]. Simvastatin (kindly provided by Merck Inc., Whitehouse Station, NJ) was activated by heating in ethanolic NaOH for 2 h at 50 °C, adjusting to pH 7.2 with HCl, and diluting to create a 10 mM stock solution. The control buffer underwent the same procedure without simvastatin added. LCLs were exposed to 2 μM simvastatin or control buffer for 24 h, and total RNA was extracted as previously described [18]. PolyA-selected RNA was made into strand-specific [55] libraries for 100 or 101 bp Illumina paired-end sequencing similar to previously described [56], except that this experiment included samples from an additional 262 LCLs in 2 additional library preparation and sequencing batches.

Using Tophatv2.0.4 [57], sequences were aligned to the human (hg19) and Epstein-Barr virus (EBV; NC_007605) genomes with Ensembl v67 [58] and EBV [59] transcriptome annotations, allowing 4 mismatches per read. Duplicate fragments were removed, and samples that did not meet quality control criteria (described previously [56]) were excluded. Fragments aligning to annotated genes were counted using HTSeq [60] and adjusted for library size and variance stabilized (roughly a log_2_ transformation) using DESeq2 [61]. Gene expression changes (deltas) were calculated by subtracting endogenous from statin-treated variance stabilized expression levels.

For downstream eQTL analyses, gene expression levels or changes were adjusted for potential confounders using probabilistic estimation of expression residuals (PEER) [62]. For endogenous, statin-treated, and average expression levels, we used K = 40 hidden factors and a mean expression covariate. This appeared to be a sufficient number of hidden factors, because very little expression level variance was explained by factors 13–40. For statin-induced gene expression changes, we used K = 6 and 7 measured covariates (delta fraction of fragments aligned, delta fraction of duplicate fragments, delta fraction of ribosomal fragments, delta fraction of EBV fragments, delta fraction of fragments aligning to annotated mRNA transcripts, delta fraction of fragments aligning to the annotated strand, and delta 5′➔3′ bias). Additional hidden factors for this analysis would have been redundant, since pilot analyses indicated that any additional hidden factors were strongly correlated to one of the first six (Spearman correlation > 0.9, *p* < 1 × 10^− 74^). Since the changes in gene expression were calculated between samples from genetically identical cell lines processed in the same experimental batches, it is expected that fewer hidden factors were necessary for the gene expression change analysis compared to the expression level analyses. For *OAS1* splicing analyses, junction-spanning sequence fragments were quantified using Leafcutter [63].

### eQTL analyses

PEER normalized gene expression levels or changes were tested for association with well-imputed (imputation Rsq ≥ 0.5) common (≥3% MAF in European Americans or ≥ 5% MAF in African Americans) genetic variants within 1 Mb of the transcription start site (in cis) using FastQTL [22] in the European and African American subsets separately. Sex and the first three ancestry principal components were included as covariates, and gene expression phenotypes were normalized prior to analysis. To adjust for testing multiple variants per gene, 100 to 100,000 adaptive permutations were conducted per gene for endogenous, statin-treated, and average eQTL analyses, while 100 to 1,000,000 were conducted for differential eQTL analyses. The most significant eQTL association per gene was retained, and these *p*-values were false discovery rate (FDR)-adjusted to account for the number of genes tested.

eQTL meta-analyses incorporating results from both European and African Americans were conducted in METAL [23], using p-value, direction of effect, and sample size as input. Heterogeneity between ethnic subsets was also evaluated. A conservative threshold of 5.78 × 10^− 9^ was used for significance of the deQTL meta-analysis based on ≤13,841 genes tested and 625 independent markers per 2 Mb of genome. (One million independent markers per genome divided by 3200 Mb/genome would estimate 312.5 independent markers per Mb.)

## Supplementary information

**Additional file 1: Table S1.** Lead endogenous eQTLs per gene in LCLs from European ancestry subset.

**Additional file 2: Table S2.** Lead endogenous eQTLs per gene in LCLs from African American ancestry subset.

**Additional file 3: Table S3.** Lead LCL endogenous eQTLs per gene from meta-analysis with *p* < 0.00001.

**Additional file 4: Table S4.** Lead statin-treated eQTLs per gene in LCLs from European ancestry subset.

**Additional file 5: Table S5.** Lead statin-treated eQTLs per gene in LCLs from African American ancestry subset.

**Additional file 6: Table S6.** Lead statin-treated LCL eQTLs per gene from meta-analysis with *p* < 0.00001.

**Additional file 7: Table S7.** Lead European ancestry eQTL for average of endogenous and statin-treated LCL gene expression levels.

**Additional file 8: Table S8.** Lead African American ancestry eQTL for average of endogenous and statin-treated LCL gene expression levels.

**Additional file 9: Table S9.** Lead eQTL for average of endogenous and statin-treated LCL gene expression levels from meta-analysis with *p*<0.00001.

**Additional file 10: Table S10.** eQTL relationships in public datasets of our deQTL associations.

## Data Availability

CAP subject genotype and LCL RNA-seq data used in this study are in the database of Genotypes and Phenotypes (dbGaP) under phs000481.v3.p2. All eQTL results are being deposited into the genome-wide repository of associations between SNPs and phenotypes (GRASP) database [64].

## Abbreviations

deQTL: Differential expression quantitative trait locus
LCL: Lymphoblastoid cell line
CAP: Cholesterol and Pharmacogenetics
eQTL: Expression quantitative trait locus
LDL-C: Low-density lipoprotein cholesterol
GWAS: Genome-wide association study
GTEx: Genotype-Tissue Expression
FDR: False discovery rate
PEER: Probabilistic estimation of expression residuals
EBV: Epstein-Barr virus
TBC1D4: TBC1 domain family member 4
MDGA1: MAM domain containing glycosylphosphatidylinositol anchor 1
CHI3L2: Chitinase 3 like 2
OAS1: 2′-5′-oligoadenylate synthetase 1
GATM: Glycine amidinotransferase
ASNSD1: Asparagine synthetase domain containing 1
GLUL: Glutamate-ammonia ligase
TDRD12: Tudor domain containing 12
PPIP5K2: Diphosphoinositol pentakisphosphate kinase 2
OAS3: 2′-5′-oligoadenylate synthetase 3
SERPINB1: Serpin family B member 1
ANKDD1A: Ankyrin repeat and death domain containing 1A
DTD1: D-aminoacyl-tRNA deacylase 1
CYFIP2: Cytoplasmic FMR1 interacting protein 2
GSDME: Gasdermin E
DFNA5: Deafness, autosomal dominant 5
